# Primary prescription adherence for obstructive lung disease in a primary care population

**DOI:** 10.1186/s13223-021-00540-7

**Published:** 2021-06-12

**Authors:** Alexander G. Singer, Alan Katz, Lisa LaBine, Lisa M. Lix, Marina Yogendran, Ian Sinha, Elissa M. Abrams

**Affiliations:** 1 Department of Family Medicine, University of Manitoba, Winnipeg, MB Canada; 2 Departments of Family Medicine and Community Health Sciences, University of Manitoba, Winnipeg, MB Canada; 3 Department of Community Health Sciences, University of Manitoba, Winnipeg, MB Canada; 4 Manitoba Centre for Health Policy, University of Manitoba, Winnipeg, MB Canada; 5 Division of Child Health, University of Liverpool, Liverpool, UK; 6 Department of Pediatrics, Section of Allergy and Clinical Immunology, University of Manitoba, FE125-685 William Avenue, Winnipeg, MB R3E 0Z2 Canada

**Keywords:** Asthma, Obstructive lung disease, Prescription adherence

## Abstract

**Background:**

The objective of this study was to determine primary prescription adherence for obstructive lung diseases (e.g., asthma, COPD) in an adult primary care patient population over a 3-year period.

**Methods:**

A retrospective analysis of electronic medical record and administrative data was performed to determine primary adherence, defined as dispensation of a new prescription within 90 days of the date the prescription was written. Multivariable logistic regression models were used to test predictors of prescription primary adherence.

**Results:**

Of 13,220 prescriptions for obstructive airway disease, 75.9% (N = 10,038) were filled. In multivariate analysis, depression, certain age groups (18–44 years), higher income quartile were associated with reduced prescription adherence. However, 1–2 ER visits in the previous year (compared to no ER visits), number of ambulatory visits in the previous year, and number of hospitalizations in the previous year, did not increase the likelihood of prescription adherence.

**Interpretation:**

This study provides important insights about factors associated with prescription nonadherence and is the first study examining primary medication adherence with medications for obstructive lung disease in adults, providing indications of prescription nonadherence patterns among a broad population.

## Introduction

Obstructive lung diseases [e.g., asthma, chronic obstructive pulmonary disease (COPD)] are leading causes of morbidity/mortality, and one of the ten leading causes of disability-adjusted life years in adults [1]. There is consistent evidence that medication adherence improves outcomes for asthma and COPD [2, 3]. Primary prescription adherence and its associated factors have not been previously studied. The objective of this study was to determine primary prescription adherence for obstructive lung diseases (e.g., asthma, COPD) in an adult primary care patient population over a 3-year period.

## Methods

A retrospective analysis was performed on data from the Manitoba Primary Care Research Network (MaPCReN), a repository of de-identified primary care electronic medical record (EMR) data. MaPCReN includes 44 primary care clinics (241 providers, over 200,000 patients age 18 years and older linked to the Manitoba Health Insurance Registry). Prescriptions written from April 1, 2012–December 31, 2015 were linked to Manitoba’s Drug Program Information Network (DPIN) data for medications that belong to the Anatomical Therapeutic Chemical (ATC) classification for medications for obstructive airway diseases.

Outcome was primary adherence, defined as dispensation of a new prescription (i.e., not dispensed within the prior 365 days) within 90 days of the date the prescription was written. Exclusion criteria included hospital admission or death within 90 days of the prescription date.

Multivariable logistic regression analysis was used to test predictors of medication primary adherence, including: patient age, sex, income quintile [measure of area-level socioeconomic status from lowest (Q1) to highest (Q5) income, or missing], healthcare use [diagnoses, hospitalizations, primary care visits, emergency department (ED) visits, prescriptions dispensed], and the Charlson Comorbidity Index (a composite medical complexity score that predicts mortality) [4]. To calculate analysis of number of unique prescriptions per patient, prescriptions were counted within the 4th level of ATC (i.e., therapeutic subgroup or drug class). The specific comorbidity of interest (depression) was defined based on a validated case definition [5]. Analyses were conducted using SAS Version 9.4.

## Results

Table 1 describes the demographics and adherence data of the patient population. The majority of patients were female (61.4%; N = 8115). The most common age groups were 18–44 years (37.6%; N = 4979) and 45–64 years (38.2%; N = 5042).

**Table 1:** Cohort Characteristics

Variable	Filled n (%)	Unfilled n (%)	OR (95% CI)
Sex			
Male	3880 (29.4)	1225 (9.3)	Ref
Female	6158 (46.6)	1957 (14.8)	0.99 (0.91, 1.07)
Age			
18–44	3718 (28.1)	1261 (9.5)	**0.84 (0.72, 0.98)**^a^
45–64	3871 (29.3)	1171 (8.9)	0.93 (0.80, 1.08)
65–74	1319 (10.0)	445 (3.4)	**0.83 (0.70, 0.98)**
75 +	1130 (8.6)	305 (2.3)	Ref
Total	**10,038**	**3182**	

Demographic characteristics of study cohort and their association with adherence from multivariable logistic regression (note: estimates statistically significant at alpha = 0.05)

^a^Statistically significant estimates in boldface font

During the study period, there were 13,220 prescriptions classified as drugs for obstructive airway disease (ATC code RO3), of which 75.9% (N = 10,038) were filled within 90 days. In multivariate analysis, depression was associated with a decreased odds of prescription adherence (OR 0.87; 95% CI 0.76–0.98) as were certain age groups (18–44 years; OR 0.84; 95% CI 0.72–0.98 and 65–74 years; 95% CI 0.70–0.98) (Table 2). Income quintile also affected likelihood of prescription adherence, with increased likelihood of filling prescriptions among lower income quintiles. Documentation in the chart of 3 or more ED visits in the prior year (compared to no ED visits (OR 1.36; 95% CI 1.01–1.84), ≥ 6 prescriptions filled in the previous year (compared to 0–2 prescriptions; OR 1.22; 95% CI 1.07–1.38) and greater comorbidity increased the likelihood of prescription adherence. However, 1–2 ER visits in the previous year (compared to no ER visits), number of ambulatory visits in the previous year, and number of hospitalizations in the previous year, did not increase the likelihood of prescription adherence.

**Table 2:** Factors associated with prescription nonadherence

Variable	Filled n (%)	Unfilled n (%)	OR (95% CI)
Age			
18–44	3718 (28.1)	1261 (9.5)	**0.84 (0.72, 0.98)**
45–64	3871 (29.3)	1171 (8.9)	0.93 (0.80, 1.08)
65–74	1319 (10.0)	445 (3.4)	**0.83 (0.70, 0.98)**
75 +	1130 (8.6)	305 (2.3)	Ref
Income quintile^a^			
NF	281 (2.1)	89 (0.7)	1.14 (0.88, 1.47)
Q1	1749 (13.2)	526 (4.0)	**1.20 (1.04, 1.37)**
Q2	2093 (15.8)	586 (4.4)	**1.30 (1.14, 1.48)**
Q3	2068 (15.6)	589 (4.5)	**1.28 (1.13, 1.46)**
Q4	2077 (15.7)	737 (5.6)	1.04 (0.92, 1.17)
Q5	1770 (13.4)	655 (5.0)	Ref
Charlson comorbidity index^b^			
0	5873 (44.4)	1762 (13.3)	Ref
1–2	3663 (27.7)	1232 (9.3)	0.82 (0.75, 0.90)
3 +	502 (3.8)	188 (1.4)	0.66 (0.54, 0.80)
Depression diagnosis			
No	8922 (67.5)	2795 (21.1)	Ref
Yes	1116 (8.4)	387 (2.9)	**0.87 (0.76, 0.98)**
Number of hospitalizations in prior 1 year			
0	9030 (68.3)	2882 (21.8)	Ref
1–2	917 (6.9)	273 (2.1)	1.09 (0.93, 1.27)
3 +	91 (0.7)	27 (0.2)	1.08 (0.69, 1.70)
Number of ambulatory visits in prior 1 year			
0	876 (6.6)	255 (1.9)	Ref
1–2	2018 (15.3)	660 (5.0)	0.90 (0.76, 1.06)
3–9	4925 (37.3)	1568 (11.9)	0.90 (0.76, 1.06)
10 +	2219 (16.8)	699 (5.3)	0.86 (0.71, 1.04)
Number of prescriptions at 4th level of ATC in prior 1 year			
0–2	3399 (25.7)	1140 (8.6)	Ref
3–5	2813 (21.3)	910 (6.9)	1.08 (0.96, 1.20)
6 +	3826 (29.0)	1132 (8.6)	**1.22 (1.07, 1.38)**
Number of ED visits in prior 1 year			
0	8752 (66.2)	2787 (21.1)	Ref
1–2	1028 (7.8)	338 (2.6)	0.95 (0.83, 1.08)
3 +	258 (2.0)	57 (0.4)	**1.36 (1.01, 1.84)**
Total	**10,038**	**3182**	

Statistically significant estimates in boldface font

^a^Manitoba centre for health policy. Concept: income quintiles—child health income quintiles. http://mchp-appserv.cpe.umanitoba.ca/viewConcept.php?printer=Y&conceptID=1161 Last updated: 2019-04-24. Accessed 27 Oct 2020

^b^Manitoba centre for health policy. Concept: charlson comorbidity Index. http://mchp-appserv.cpe.umanitoba.ca/viewConcept.php?conceptID=1098 Last updated: 2019-01-17. 27 Oct 2020

## Discussion

To our knowledge this is the first study examining primary prescription adherence with medications for obstructive lung disease in adults. It demonstrates robust primary adherence at 76%, which is higher than secondary adherence in adults with asthma of between 30 and 70%, and with COPD of between 40 and 60% [2]. Poor medication adherence in adults with asthma and COPD has been associated with increased disease morbidity, mortality, higher healthcare costs, and reduced quality of life [2, 3, 6].

Depression was associated with reduced prescription adherence, in keeping with previous studies in both asthma and COPD [3, 7]. Studies have documented that prevalence of depression is twice as high among adults with obstructive pulmonary disease [8]. Depression has been independently linked with poorer health outcomes and reduced quality of life, both of which could compound prescription nonadherence [3].

Certain age groups were associated with prescription non-adherence (18–44 years and 65–74 years). Further studies are required to validate these findings to determine if this association is consistent in other settings.

Previous studies have documented a higher income quintile/increased socioeconomic status to increase the rate of medication adherence [9]. In contrast, this study demonstrated lower income quintiles to be associated with increased prescription adherence. While typically lower income reduces access to health care and affordability of medications, Manitoba has means tested pharmaceutical coverage. Our data did not include drug insurance claims data, so we were unable to fully describe the impact of medication coverage.

Medical complexity increased the risk of prescription nonadherence. Medical comorbidities is relatively consistently associated with medication nonadherence [6, 7, 9]. Possible solutions to improve prescription adherence in medically complex patients include ensuring patients understand the importance of not treating one condition at the detriment of another, and improved patient education both in a medical facility and in pharmacies [9].

While ≥ 3 ED visits in the prior year increased the likelihood of prescription adherence, other measures of possible lung disease severity including 1–2 ED visits in the previous year, number of ambulatory visits in the previous year, and number of hospitalizations in the previous year did not increase prescription adherence. Medication adherence in asthma and COPD have been associated with a reduced risk of exacerbations [2, 3], and reverse causation is possible whereby reduced prescription adherence led to an increased likelihood of exacerbations, although the reasons for the ED visits and hospitalizations were not captured in the database. Another possible explanation is poor perception of disease, higher medical complexity, or reduced access to medications, among those at higher risk.

A limitation of our retrospective analysis is that our study population is from a single Canadian province, which may affect external validity. We did not capture medications prescribed by specialists (e.g., pulmonologists), from whom primary adherence rates may be different. As a result of these limitations, it is possible that primary adherence is underestimated. Race/ethnicity, smoking status, health literacy, and level of education were not measured and are possible confounders. A further limitation is that the data cannot separate out whether this medication was prescribed for COPD versus asthma, or for another reason (such as viral infection), nor can we ascertain disease severity and its impact on adherence. We also cannot ascertain whether samples of medications may have been provided. In addition, some who do not meet criteria for pharmaceutical coverage may still find the cost of medications to be a significant barrier to adherence.

In conclusion, this study provides important insights about factors associated with prescription nonadherence and is the first study examining primary medication adherence with medications for obstructive lung disease in adults.

## Data Availability

The datasets generated and/or analysed during the current study are not publicly available.

## Abbreviations

COPD: Chronic obstructive pulmonary disease
ED: Emergency department
EMR: Electronic medical record
